# Exploring the neuroprotective effects of fenchone against ICV‐STZ‐induced Sporadic Alzheimer’s disease in rats: Investigating neuroinflammation, oxidative stress and mitochondrial dysfunction as potential mechanisms

**DOI:** 10.1002/alz.091350

**Published:** 2025-01-03

**Authors:** Mini Mini

**Affiliations:** ^1^ UIPS, Panjab University, Chandigarh, Chandigarh India

## Abstract

**Background:**

Sporadic Alzheimer’s disease (sAD) is the most prevalent type of dementia, characterized by progressive neurodegeneration. Intracerebroventricular (ICV) administration of streptozotocin (STZ) serves as a model for sAD, inducing neurodegeneration through oxidative stress, apoptotic damage, mitochondrial dysfunction, and neuroinflammation. Fenchone, a monoterpenoid, has been reported to possess neuroprotective properties by attenuating oxidative stress, acetylcholinesterase activity, mitochondrial dysfunction and neuroinflammation. This study aimed to explore the neuroprotective potential of fenchone in ICV‐STZ‐induced sAD and its associated pathology in wistar rats.

**Method:**

To assess the neuroprotective potential of fenchone (20, 40, and 80 mg/kg) and galantamine (2 mg/kg) were given orally in ICV‐STZ‐induced wistar rats for 21 days. Behavioral assessments, including locomotor activity, Morris water maze, and novel object recognition, were conducted. Subsequently, rats were sacrificed on the 21st day, hippocampus and pre‐frontal cortex were prepared for the quantification of oxidative stress, apoptotic markers, mitochondrial complex activity, acetylcholinesterase activity, and neuroinflammatory markers.

**Result:**

*In‐vivo*, studies revealed that fenchone treatment ameliorated ICV‐STZ‐induced cognitive deficits in the Morris water maze and novel object recognition tests. Furthermore, fenchone arrested oxidative stress (glutathione (GSH), catalase, superoxide dismutase (SOD), and lipid peroxidation (LPO)), decreased apoptotic damage (caspase 3) and neuroinflammation (TNF‐α and IL‐6) in the cortex and hippocampus regions of the brain. Additionally, normalization of mitochondrial enzyme complexes and acetylcholinesterase activities was observed.

**Conclusion:**

This study demonstrates the neuroprotective effects of fenchone in the ICV‐STZ rat model, alleviating oxidative stress, apoptotic damage, reversing mitochondrial dysfunction, acetylcholinesterase activity, and neuroinflammation in brain.

